# Concentration- and Time-Dependent Effects of Benzalkonium Chloride in Human Lung Epithelial Cells: Necrosis, Apoptosis, or Epithelial Mesenchymal Transition

**DOI:** 10.3390/toxics8010017

**Published:** 2020-03-02

**Authors:** Sou Hyun Kim, Doyoung Kwon, Seunghyun Lee, Seung Won Son, Jung-Taek Kwon, Pil-Je Kim, Yun-Hee Lee, Young-Suk Jung

**Affiliations:** 1 Lab of Molecular Toxicology, College of Pharmacy, Pusan National University, Busan 46241, Korea; 2 Department of Cellular and Molecular Pharmacology, University of California San Francisco, San Francisco, CA 94158, USA; 3 Risk Assessment Division, Environmental Health Research Department, National Institute of Environmental Research, Incheon 22689, Korea; 4 College of Pharmacy and Research Institute of Pharmaceutical Sciences, Seoul National University, Seoul 08826, Korea

**Keywords:** benzalkonium chloride, lung epithelial cells, cytotoxicity, endoplasmic reticulum stress, epithelial–mesenchymal transition

## Abstract

Benzalkonium chloride (BAC), an antimicrobial agent in inhalable medications and household sprays, has been reported to be toxic to pulmonary organs. Although cell membrane damage has been considered as the main cytotoxic mechanism of BAC, its concentration- and time-dependent cellular effects on lung epithelium have not been fully understood. In the present study, human lung epithelial (H358) cells were exposed to 0.2–40 μg/mL of BAC for 30 min or 21 days. Cell membranes were rapidly disrupted by 30 min exposure, but 24 h incubation of BAC (4–40 μg/mL) predominantly caused apoptosis rather than necrosis. BAC (2–4 μg/mL) induced mitochondrial depolarization, which may be associated with increased expression of pro-apoptotic proteins (caspase-3, PARP, Bax, p53, and p21), and decreased levels of the anti-apoptotic protein Bcl-2. The protein expression levels of IRE1α, BiP, CHOP, and p-JNK were also elevated by BAC (2–4 μg/mL) suggesting the possible involvement of endoplasmic reticulum stress in inducing apoptosis. Long-term (7–21 days) incubation with BAC (0.2–0.6 μg/mL) did not affect cell viability but led to epithelial-mesenchymal transition (EMT) as shown by the decrease of E-cadherin and the increase of N-cadherin, fibronectin, and vimentin, caused by the upregulation of EMT transcription factors, such as Snail, Slug, Twist1, Zeb1, and Zeb2. Therefore, we conclude that apoptosis could be an important mechanism of acute BAC cytotoxicity in lung epithelial cells, and chronic exposure to BAC even at sub-lethal doses can promote pulmonary EMT.

## 1. Introduction

Benzalkonium chloride (BAC), a mixture of n-alkylbenzyldimethylammonium chlorides, is an antimicrobial agent used in agricultural, medical, and household products as a disinfectant, an antiseptic, and a preservative, respectively [1]. BAC has been known to have potent bactericidal/fungicidal properties due to its ability to destroy the cell membrane [2,3,4].

In clinical studies, the adverse effects of BAC on the human body, including the skin, eye, nasal, and pulmonary tissues, have been reported [5,6,7,8,9]. Among these, the pulmonary effects of BAC have been evaluated, since this preservative is found in inhalable medications such as nasal sprays, nebulizers, and inhalers for patients with nasal congestion, allergic rhinitis, asthma, and chronic obstructive pulmonary diseases (COPD) [6,7]. Moreover, BAC is included in household products that use sprays and atomizers, such as bathroom cleaners, deodorants, and air fresheners, leading to BAC inhalation by humans [1]. BAC in nebulized bronchodilators has been shown to cause bronchoconstriction in asthmatic and COPD patients [6,7,10]. Especially, 17 in 28 asthmatic patients showed at least 20% decrease of 1-s forced expiratory volume (PD_20_FEV_1_) after the inhalation of anti-asthma respirator solutions containing BAC (0.35 to 5.55 µmol) [10]. Animal studies have demonstrated that BAC inhalation induces pulmonary irritation, inflammation, and damages the blood-air barrier in rodents [11,12,13,14,15]. Several mechanisms of BAC cytotoxicity, such as cell membrane disruption, oxidative stress, and DNA damage have been proposed in in vitro studies using human lung cell lines [14,16]. Nevertheless, the exact cellular events in BAC-induced lung injury have not been fully understood.

Necrosis and apoptosis are two major death fates of cells exposed to cytotoxic stimuli [17,18]. Necrosis is an uncontrolled form of cell death characterized by the swelling of cellular organelles and rupture of the plasma membrane. In contrast, apoptosis is a programmed and self-destructive death of cells that undergo cellular shrinkage, DNA fragmentation, and apoptotic body formation [17,18]. In general, necrosis is accidently caused by acute and extreme stimuli, whereas apoptosis occurs when the cells are chronically exposed to various stimuli at low doses [17,19]. In ocular cells, it has been observed that BAC induces concentration-dependent necrotic or apoptotic cell death [20,21]. Highly toxic concentrations of BAC rapidly induce necrosis in human conjunctival cells, while lower concentrations promote apoptosis in a delayed manner [19,21]. Specifically, corneal and conjunctival apoptosis have been suggested to be important effects of BAC-induced eye injuries [22,23]. In lung cells, however, the dose-dependent necrotic and/or apoptotic effects of BAC have not been identified. Moreover, the apoptogenic effect of BAC on pulmonary cells and the mechanism has been still largely uncertain.

Epithelial-mesenchymal transition (EMT) is a cellular process in which epithelial cells gradually transform into mesenchymal-like cells acquiring fibrotic and migratory features [24,25]. Cells undergoing EMT synthesize extracellular matrix components (ECM), and lose their epithelial characteristics such as apical-basal polarity and cell-cell adhesion. Thus, EMT can cause tissue fibrogenesis and cancer metastasis [24,25]. Pulmonary EMT has been found in lung diseases such as asthma, COPD, idiopathic pulmonary fibrosis, and lung cancer [24,25]. Inhalable toxicants such as cigarette smoke, airborne particles, and chemotherapeutics have been shown to cause lung fibrosis via the induction of EMT [26,27,28]. Repeated inhalation of BAC has been reported to induce bronchioalveolar fibrosis in rats [15]. However, the involvement of EMT in BAC-promoted pulmonary fibrosis remains unknown.

The purpose of this study was to evaluate the concentration- and time-dependent effects of BAC in lung epithelial cells since various concentrations of BAC are included in inhalable medications (0.003%–0.12% of BAC) and household sprays (0.01%–1.5% of BAC). Moreover, we were interested in evaluating the long-term pulmonary influence of BAC at safe doses since this substance can be repeatedly and chronically inhaled by patients or consumers. Human bronchioalveolar epithelial (H358) cells were incubated with various concentrations (0.2–40 μg/mL) of BAC for 21 days, and the cellular responses were monitored.

## 2. Materials and Methods

### 2.1. Cell Culture

NCI-H358 human non-small cell lung cancer cells (Korea Cell Line Bank, Seoul, Korea) were cultured routinely in RPMI-1640 medium (Hyclone, Logan, UT, USA) supplemented with 10% fetal bovine serum (FBS, Hyclone), 2 mM glutamine (Sigma-Aldrich, St. Louis, MO, USA), 100 U/mL penicillin (Hyclone), and 100 μg/mL streptomycin (GenDEPOT, Barker, TX, USA) at 37 °C with 5% CO_2_ in a humidified atmosphere.

### 2.2. Cell Viability Assay

Cell viability was examined by MTT assay as described previously [29]. Briefly, the cells in a 96-well plate were treated with different concentrations of BAC and then incubated with 0.5 mg/mL MTT at 37 °C for 1 h. The absorbance of the converted dye was measured at 540 nm using the MULTISKAN GO reader (Thermo Scientific, Sunnyvale, CA, USA), and the results were expressed as a percentage of viable BAC-treated cells normalized to the percentage of untreated cells.

### 2.3. LDH Leakage Assay

Cell cytotoxicity was measured using the DG-LDH500 kit (DoGenBio, Seoul, Korea). The cells were grown to 90% confluency, treated with different concentrations of BAC for 30 min. The 10 μL supernatant was collected and mixed with LDH Reaction Mixture (100 μL). After incubation for 30 min at room temperature (RT), the absorbance was measured at 450 nm using MULTISKAN GO reader (Thermo Scientific).

### 2.4. Annexin V Assay and PI Staining Analysis of Apoptosis

Apoptosis was determined according to the instruction of Annexin V-FITC Apoptosis Detection Kit (BD Biosciences, Bedford, MA, USA) as we reported previously [29]. The stained cells by annexin V-FITC and propidium iodide (PI) were analyzed by Becton Dickinson FACSscan flow cytometer (BD Biosciences, San Jose, CA, USA) and BD FACSDiva software (BD Biosciences).

### 2.5. Caspase-3 Activity

The activity of caspase-3 was determined in the same manner as we reported [30]. The fluorescence (Ex380, Em460) by cleavage of substrates Ac-DEVD-AMC was measured using Promega Glomax microplate reader (Promega, Madison, WI, USA).

### 2.6. Examination of Mitochondrial Membrane Potential (MMP, ∆ψ)

The mitochondrial membrane potential (MMP, ∆ψ) was detected by staining of tetraethylbenzimidazolylcarbocyanine iodide (JC-1) according to the protocol we reported [29]. Stained cells were subjected to FACSscan flow cytometer and BD FACSDiva software (BD Biosciences)

### 2.7. Western Blot Analysis

Protein samples were obtained from cells lysed with chilled ProEXTM CETi protein extract solution (Translab, Daejeon, Korea). Denatured samples were separated by sodium dodecyl sulfate (SDS)-polyacrylamide gel electrophoresis. Then, the proteins were transferred onto nitrocellulose (NC) membranes (Bio-Rad, Hercules, CA, USA) [30]. The membranes were incubated overnight at 4 °C with primary antibodies such as anti-PARP, anti-cleaved caspase-3, anti-Bip, anti-CHOP, anti-p-JNK, anti-E-cadherin, anti-N-cadherin (Cell Signaling Technology, Beverly, MA, USA), anti-p53, anti-p21, anti-Bax, anti-Bcl2, anti-GAPDH, anti-β-actin (Santa Cruz Biotechnology, Santa Cruz, CA, USA) and anti-IRE1α (Abcam, Cambridge, MA, USA). After the membranes were incubated for 1 h with the appropriate horseradish peroxidase-conjugated secondary antibodies, the proteins were detected using EZ-Western Lumi Pico (DoGenBio).

### 2.8. Immunofluorescence Staining

Cells were fixed with 4% paraformaldehyde (PFA) for 20 min and permeabilized in 0.2% Triton X-100 for 10 min at RT. Cells were incubated with Alexa-488-conjugated E-cadherin (1:500, Cell Signaling Technology, Danver, MA, USA) and Alexa-555-conjugated vimentin antibodies (1:100, Cell Signaling Technology) at 4 °C overnight and staining was detected by green and red fluorescence, respectively. Nuclei were stained blue using DAPI. The cells were examined under a confocal microscope.

### 2.9. Real-Time Reverse Transcription-Polymerase Chain Reaction (RT-PCR)

Isolation of total RNA using the Direct-zol™ RNA kit (Zymo Research, Orange, CA, USA) and synthesis of cDNA using the iScript™ cDNA Synthesis kit (Bio-Rad, Hercules, CA, USA) followed the manufacturer’s protocol. The transcripts were amplified using the SensiFAST SYBR qPCR mix (Bioline, London, UK) [31]. Table 1 shows the primers used for amplification.

### 2.10. Cell Migration Assay

We utilized Costar (Cambridge, MA, USA) plates containing polycarbonate filter inserts with a pore size of 8 µm. NCI-H358 cells were placed in the upper chamber containing serum-free medium, while the lower chamber contained medium with 10% FBS. The transwell chambers were incubated for 16 h and the number of cells that moved to the bottom of the insert were fixed, stained with crystal violet, and counted to determine the relative migration.

### 2.11. Statistical Analysis

Results were expressed as mean ± SD and were analyzed by two-tailed Student’s t-test (statistical significance at *p* < 0.05).

## 3. Results

### 3.1. BAC-Induced Cytotoxicity in Lung Epithelial Cells

**Figure 1:**
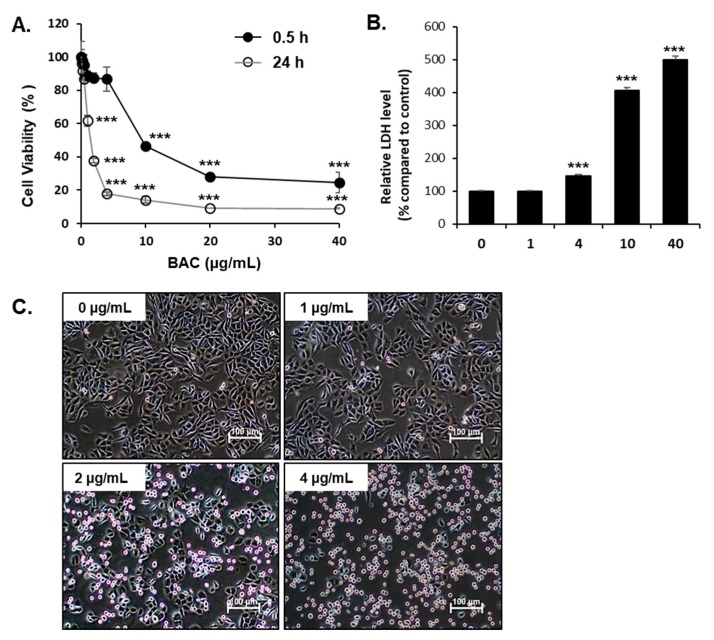
Benzalkonium chloride (BAC) cytotoxicity in human lung epithelial (H358) cells. (**A**) Examination of cell viability using MTT assay in cells after 30 min or 24 h of BAC exposure. (**B**) Determination of lactate dehydrogenase (LDH) leakage from cells after 30 min of BAC exposure. The results are expressed as a percentage compared to untreated cells and each value represents the mean ±SD. ***Significantly different from the corresponding untreated cells (Student’s t-test, *p* < 0.001). (**C**) Morphological changes in H358 cells treated with BAC for 24 h. Scale bar, 100 µm.

BAC induced concentration- and time-dependent cytotoxicity in H358 cells (Figure 1). At higher concentrations (>10 μg/mL) of BAC, cell viability was dramatically decreased after 30 min incubation (Figure 1A). The lactate dehydrogenase (LDH) level in the medium, which indicates cell membrane damage, was significantly increased after 30 min exposure to BAC (>4 μg/mL) (Figure 1B). At 24 h treatment, 1–40 μg/mL of BAC decreased cell viability, and >80% of the cells were dead at >4 μg/mL of BAC (Figure 1A,C). The IC50 of BAC at 30 min and 24 h incubation periods were 7.1 μg/mL and 1.5 μg/mL, respectively.

### 3.2. Induction of Necrosis and Apoptosis by BAC in Lung Epithelial Cells

**Figure 2:**
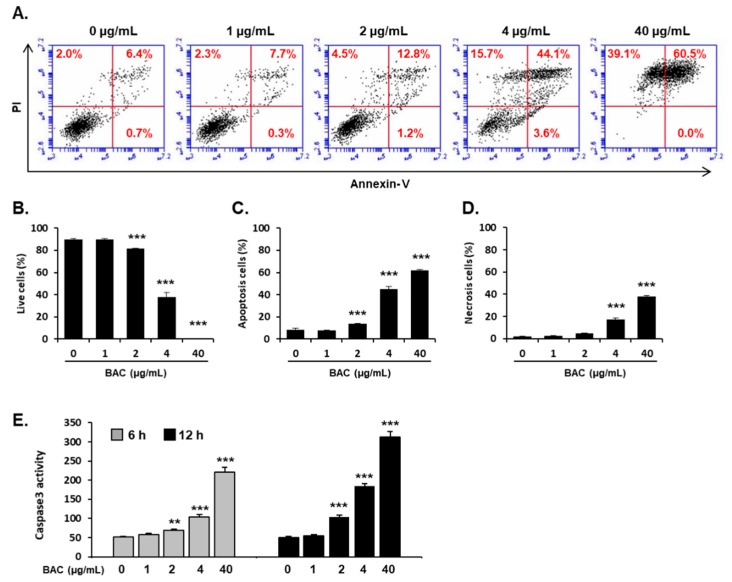
Induction of necrosis and apoptosis in H358 cells treated with BAC. Cells were treated with BAC for 24 h (**A**–**D**). (**A**) Fluorescence-activated cell sorting (FACS) analysis of propidium iodide (PI) uptake and annexinV binding in non-permeabilized cells (Lower-left, live cells; lower-right, early apoptotic cells; upper-right, late apoptotic cells; upper-left, necrotic cells). Quantification of (**B**) live cells, (**C**) apoptotic cells, and (**D**) necrotic cells. (**E**) Caspase-3 activity in the cells treated with BAC for 6 h and 12 h. The results are presented as the mean ± SD of triplicate experiments. Statistical significance was determined by Student’s t-test (**p < 0.01, ***p < 0.001, compared to untreated cells).

Since BAC treatment rapidly damaged the cell membranes (Figure 1B), we hypothesized that necrotic cell lysis could be the major cell death pathway of cells exposed to BAC at higher concentrations. Cell death processes were monitored by fluorescence-activated cell sorting (FACS) analysis using Annexin V and propidium iodide (PI) after 24 h of BAC incubation. BAC (4–40 µg/mL) increased both necrotic and apoptotic cell death in a dose-dependent manner (Figure 2A–D). However, apoptosis was predominantly induced in cells treated with cytotoxic concentrations (2–40 µg/mL) of BAC (Figure 2A–D). At the highest concentration of BAC (40 µg/mL), most of the cells were dead, and the percentage of apoptotic and necrotic cells were 60.5% and 39.1%, respectively (Figure 2A–D). To determine the effect of BAC on apoptosis in early time period, caspase-3 activity, an indicator of apoptosis, was measured at 6 h and 12 h after BAC treatment. BAC (2–40 µg/mL) increased caspase-3 activity in a dose- and time-dependent manner (Figure 2E). These results suggest that apoptosis, rather than necrosis, might be the major cell death pathway induced by BAC in lung epithelial cells.

### 3.3. Mitochondrial Depolarization and Activation of the Apoptotic Signaling Pathway by BAC

**Figure 3:**
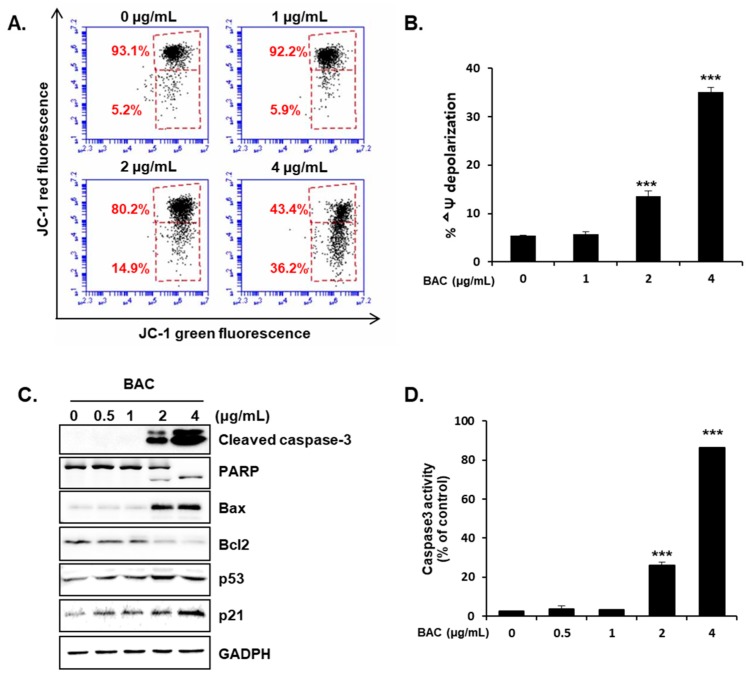
BAC-induced mitochondrial dysfunction and activation of apoptosis-related pathways in H358 cells. Cells were treated with BAC for 24 h. (**A**) Changes in mitochondrial membrane potential as observed by JC-1 staining, using fluorescence-activated cell sorting (FACS) analysis and (**B**) quantification of three independent experiments. (**C**) Protein expression of caspase-3, PARP, Bax, Bcl2, p53, and p21. GAPDH is used as a loading control. (**D**) Caspase-3 activity in H358 cells after BAC treatment for 24 h. The results are presented as the mean ± SD of triplicate experiments. Statistical significance was determined by Student’s t-test (***p < 0.001 compared to untreated cells).

To elucidate the cellular mechanism of apoptosis, mitochondrial membrane potential and apoptotic protein levels in the cells were determined after incubation of cells with BAC for 24 h. Mitochondrial depolarization, an important cause of apoptosis, was observed in 14.9% and 36.2% of cells exposed to 2 and 4 μg/mL of BAC, respectively (Figure 3A,B). The levels cellular proteins involved in the apoptotic cascade were also significantly altered by BAC treatment. The protein levels of Bcl2-associated X apoptosis regulator (Bax) and its transcriptional activator p53 were elevated, while that of the anti-apoptotic B-cell lymphoma 2 (Bcl2) was reduced by BAC (Figure 3C). Bcl2 is known to inhibit Bax which forms pores on the mitochondrial outer membrane [18,32]. Thus, the increased Bax and decreased Bcl2 expression levels could lead to decreased mitochondrial membrane potential. The protein levels and activity of cleaved caspase-3, an apoptosis executioner degrading the cellular components, were increased by BAC (2–4 μg/mL) (Figure 3C,D). The cleaved poly (ADP-ribose) polymerase (PARP) level, a biomarker of caspase activity [33,34], was also elevated (Figure 3C). Various stress signals can activate the expression of p53 which then initiates apoptosis via inducing Bax, and arrests cell cycle by elevating p21 expression [18,35]. In the present study, the increased expression levels of p53 and p21 by BAC (Figure 3C) suggest that BAC treatment induced significant cellular stress resulting in apoptosis in lung cells.

### 3.4. ER Stress Induced by BAC in Lung Epithelial Cells

**Figure 4:**
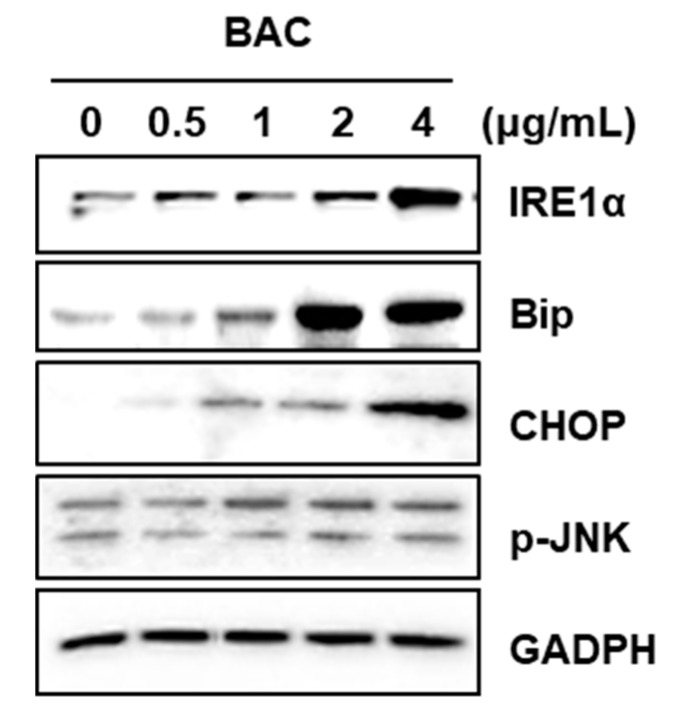
ER stress promoted by BAC in H358 cells. Western blot analysis of ER stress-related proteins was performed after BAC treatment for 24 h. GAPDH is used as a loading control.

ER stress is a cellular condition of impaired protein folding function in the ER The accumulation of unfolded/misfolded proteins in the ER activates unfolded protein response (UPR) which improves protein folding, inhibits protein synthesis, and degrades misfolded proteins for maintaining ER homeostasis. However, when the ER function cannot be restored due to severe and prolonged ER stress, apoptosis is triggered to eliminate the stressed cells, thereby protecting the organelle [36,37]. In the present study, the levels of ER stress markers, such as binding immunoglobulin protein (BiP, also called GRP78) and C/EBP homologous protein (CHOP) were increased by BAC (2–4 μg/mL) (Figure 4). The levels of the UPR sensor protein, inositol requiring enzyme 1α (IRE1α), and the downstream signaling protein, phospho-c-Jun N-terminal kinase (p-JNK), were also enhanced in BAC-treated cells (Figure 4). Since activated JNK is known to upregulate pro-apoptotic genes [38], BAC-induced ER stress could be associated with the induction of apoptosis in lung epithelial cells.

### 3.5. EMT Induced by BAC in Lung Epithelial Cells

**Figure 5:**
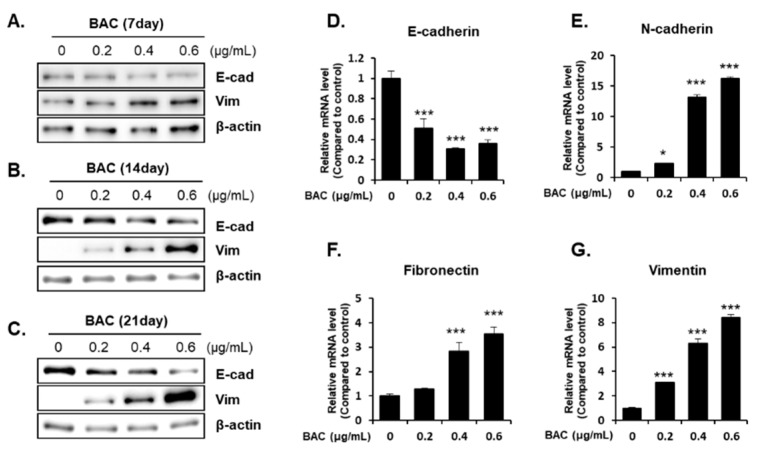
BAC-indcued epithelial–mesenchymal transition (EMT) in H358 cells**.** (**A**)–(**C**) Changes in protein levels of E-cadherin and vimentin in H358 cells exposed to BAC for 7, 14, and 21 days. β-actin is used as a loading control. mRNA levels of (**D**) E-cadherin, (**E**) N-cadherin, (**F**) fibronectin, and (**G**) vimentin after 21 days of exposure to BAC. Statistical significance was determined by Student’s t-test (*p < 0.05, ***p < 0.001, compared to untreated cells).

**Figure 6:**
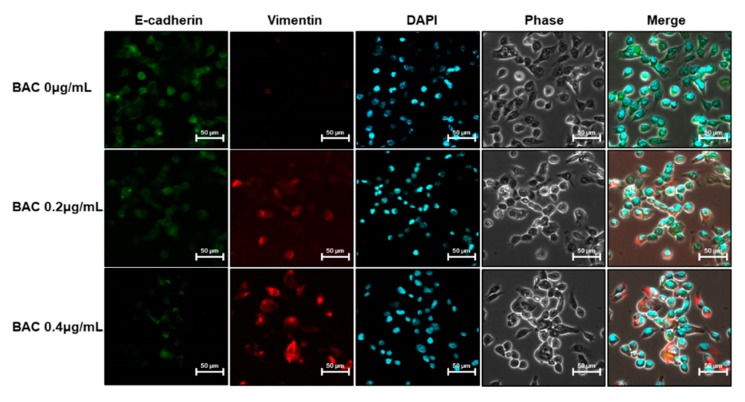
Expression levels of E-cadherin and vimentin in BAC-treated H358 cells. Immunofluorescent staining of E-cadherin (green) and vimentin (red) in H358 cells after incubation of cells with BAC for 21 days. DAPI (blue) is used for nuclear staining. Scale bar, 50 µm.

**Figure 7:**
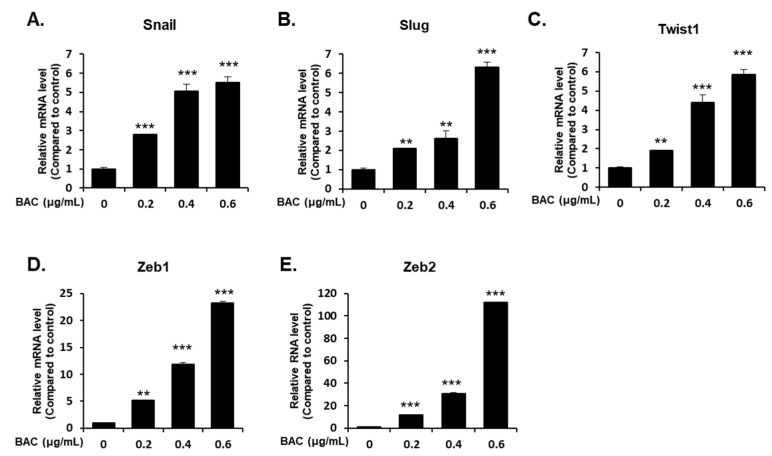
Changes in expression of EMT-inducing transcription factors. mRNA levels of (**A**) Snail, (**B**) Slug, (**C**) Twist1, (**D**) Zeb1, and (**E**) Zeb2 in H358 cells exposed to BAC for 21 days. Statistical significance was determined by Student’s t-test (**p < 0.01, ***p < 0.001, compared to untreated cells).

**Figure 8:**
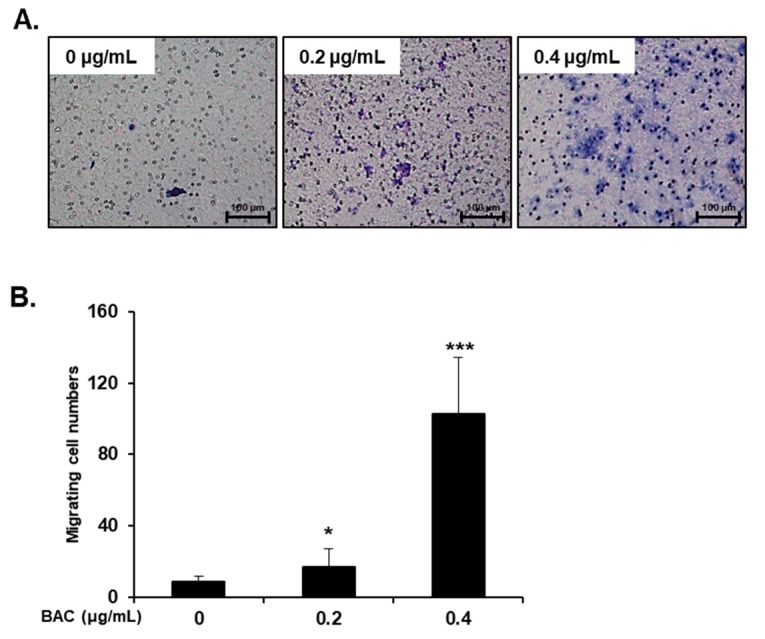
Increased cell migration by BAC treatment. H358 cells were treated with BAC for 21 days and (**A**) cell migration assay was performed for 16 h using a transwell chamber on day 22. (**B**) Migrated cells were counted and plotted as mean ± SD. Statistical significance was determined by Student’s t-test (*p < 0.05, ***p < 0.001, compared to untreated cells). Scale bar, 100 µm.

H358 cells incubated with 0.2–0.6 μg/mL of BAC did not affect the cell viability up to 21 days (data not shown). However, incubation of cells with BAC for 7, 14, and 21 days altered the levels of EMT-associated proteins in the cells. The expression of E-cadherin, which is responsible for cellular adhesion was decreased, but the expression of vimentin, a cytoskeletal component found in mesenchymal cells, was increased by BAC in a concentration- and time-dependent manner (Figure 5A–C). The decreased E-cadherin mRNA and increased vimentin mRNA might account for the altered expressions of these proteins (Figure 5D,G). Moreover, the mRNA levels of N-cadherin, a transmembrane glycoprotein that leads to cell invasion, and fibronectin, a component of the ECM, were increased by BAC (Figure 5E,F). In addition, microscopic examination after immunofluorescence staining showed that after 21 days of BAC treatment, the E-cadherin fluorescence intensity (green) in H358 cells was decreased, while that of vimentin (red) was increased in a concentration-dependent manner (Figure 6). The expression levels of EMT-inducing transcription factors, such as Snail, Slug, Twist1, Zeb1, and Zeb2, were increased by 21 days of BAC exposure in a dose-dependent manner as observed by the elevated expression levels of their mRNA (Figure 7). To confirm the induction of EMT, the migratory properties of H358 cells were examined after incubation of cells with BAC (0.2–0.4 μg/mL) for 21 days using a transwell chamber system. Significantly increased migration of cells through the membrane was observed at 16 h in BAC-treated cells compared to untreated cells (Figure 8). These results clearly show the EMT promoting effect of BAC in lung epithelial cells.

## 4. Discussion

The cytotoxicity of BAC has been reported to be due to its chemical structure, composed of NR4^+^, a positively charged nitrogen atom and long alkyl groups [2,4]. The hydrophobic carbon chains can easily penetrate the lipid bilayer of the cell membrane, and the charged nitrogen remaining at the membrane surface perturbs the charge distribution. As a result, hydrophilic voids are created, phospholipids are solubilized, and finally the membrane structure is destroyed [2,4]. In alveolar and bronchial epithelial cells, BAC (1–100 µg/mL) treatment has been shown to induce acute cytotoxicity with cell membrane disruptions [14,16]. Inhalation of BAC damages airway epithelium resulting in the release of proteins and LDH to the bronchioalveolar spaces in rodents [11,12,15]. Thus, necrotic cell lysis has been considered to be the primary consequence of BAC-induced pulmonary toxicity. In the present study, BAC treatment rapidly destroyed the cell membranes (Figure 1B). However, the major cell death pathway in H358 cells exposed to cytotoxic doses of BAC for 24 h was apoptosis rather than necrosis (Figure 2). These results suggest that apoptosis also could be an important mechanism of BAC-induced lung epithelial injury in addition to necrosis.

Apoptosis in injured lung airway has a beneficial role in maintaining organ homeostasis by removing damaged cells. However, excessive apoptosis contributes to the pathogenesis of pulmonary diseases [39]. One recently published paper shows that BAC (20–40 µg/mL) triggers apoptotic pathway via caspase-3 activation and PARP cleavage in human alveolar epithelial (A549) cells after 0.5 h, 1 h, and 4 h incubations [40]. However, other cellular events involved in apoptotic processes were not examined [40]. In the present study, BAC (2–4 µg/mL) appears to promote mitochondria-dependent apoptosis. In normal cells, the anti-apoptotic proteins Bcl2 and B-cell lymphoma-extra large (Bcl-xL) bind to Bax and Bcl2 antagonist/killer 1 (Bak) on the mitochondrial outer membrane. However, under stress conditions, Bax and Bak are separated from Bcl2 or Bcl-xL to form pores allowing the release of mitochondrial cytochrome c. The cytosolic cytochrome c triggers the activation of caspase-9 which then cleaves and activates caspase-3 to degrade cellular components [18,32]. In our results, the decreased Bcl2 and increased Bax expression levels could result in the formation of mitochondrial pores which collapse the membrane potential (ΔΨ), a driving force for ATP synthesis. Cells that are stressed by DNA damage, oxidative stress, and ER stress activate the expression of p53 which then induces the transcription of Bax [18,32,39,41], but inhibits Bcl2 expression [17]. Moreover, p53 can directly bind to the mitochondrial Bcl2 leading to the release of Bax and Bak [42,43]. In addition, p53 arrests cell cycle via inducing p21 expression thereby allowing cells to recover, but, triggers apoptosis under the severe stress conditions [35,44]. It has been reported that BAC induces DNA damage and oxidative stress in human lung epithelial cells [14,16], suggesting that these cellular stress events promoted by BAC could be possible reasons for the increased expression of p53 (Figure 3C). PARP catalyzes the transfer reaction of ADP-ribose for DNA repair, and this enzyme can be cleaved and inactivated by caspase-3 [45]. Since PARP-mediated reaction consumes ATP, the inactivation of PARP (Figure 3C) could preserve ATP for the energy-dependent apoptotic process, as observed in our study. BAC-induced apoptosis via mitochondrial depolarization, cytochrome c release, activation of caspases, and PARP cleavage has been reported in human corneal and conjunctival epithelial cell lines [21,46], suggesting similar mitochondria-involved apoptogenic effect of BAC in mammalian cells.

ER is an organelle in which newly synthesized proteins are folded by ER-resident proteins such as foldases, calreticulin, and chaperones [36,37,47]. BiP, an ER chaperone, binds to hydrophobic residues of proteins to prevent their misfolding [47]. BiP also binds to three UPR sensor proteins, IRE1α, activating transcription factor-6 (ATF6), and PKR-like eukaryotic initiation factor 2a kinase (PERK) to maintain them in an inactive state [36,37]. However, due to ER stress, BiP preferentially associates with unfolded/misfolded proteins, and releases the UPR sensor proteins thereby activating them [48]. Activated IRE1α degrades RNA to attenuate protein synthesis and mediates the translation of X box-binding protein 1 (XBP1) [36,37,47]. XBP1 then activates genes involved in ER biogenesis, ER-associated degradation, and chaperone synthesis such as synthesis of BiP [49,50]. CHOP is induced via the activation of PERK and ATF6, and the latter is also known to promote the expression of BiP [51,52]. Thus, in the present study, the increased levels of IRE1α, BiP, and CHOP proteins clearly indicate BAC-induced ER stress in lung epithelial cells.

Intensive and sustained UPR signaling can eventually trigger apoptosis [36,37,47]. CHOP is a pro-apoptotic transcription factor that inhibits Bcl2 but induces the expression of the apoptotic initiator Bim, resulting in the activation of the caspase-9 and caspase-3 axis [36,37]. Activated IRE1α forms a complex with tumor necrosis factor receptor-associated factor 2 (TRAF2) and apoptosis signal-regulating kinase1 (ASK1), and this complex subsequently phosphorylates JNK [36,37,47]. The activated JNK phosphorylates ER membrane Bcl2 resulting in the release of Bax and Bak to form pores on the ER membrane [53]. Through these pores, ER calcium is released and taken up by mitochondria leading to the depolarization of this organelle [36,37,47]. Since p-JNK is also known to activate p53 [36], the increased levels of IRE1α and p-JNK by BAC (Figure 4) indicate the possibility of ER stress-mediated apoptosis. Moreover, the elevated Bax and reduced Bcl2 protein levels in BAC-treated cells (Figure 3C) could possibly cause pore formation on the ER membrane. Previously, BAC-exposed zebrafish (Danio rerio) eleuthero-embryos and liver cells showed activated gene expression of ATF6, BiP, CHOP, and XBP1 resulting in apoptosis via the increased expression of Bax, caspase-9 and p53 [54]. Thus, in our study, BAC-promoted ER stress could be directly associated with lung epithelial apoptosis. Nevertheless, the mechanism by which BAC induced ER stress in cells, and whether ER stress was the critical cause of apoptosis remains unclear.

EMT has been observed in wounded and inflamed airway epithelia [55,56]. The potent inducer of EMT is transforming growth factor-β (TGF-β), which is secreted by inflammatory cells into the bronchioalveolar space [55,56]. This cytokine binds to epithelial transmembrane TGF-β receptors that phosphorylate Smad2 or Smad3 to form a complex with Smad4. This complex translocates into the nucleus to express EMT-inducing transcription factors, such as Snail, Slug, Twist1, Zeb1, and Zeb2, which are responsible for the downregulation of E-cadherin, and the upregulation of N-cadherin, fibronectin, vimentin, and collagen [55,56,57]. Cells undergoing EMT break down tight junctions and lose cell-cell adhesion due to loss of E-cadherin expression and gain migratory potential due to increased N-cadherin expression [56,57]. Acquiring motility and a flat shape have beneficial roles in tissue repair, since the cells can migrate to the wounded airway epithelium and spread out for reestablishing the intact barrier function [56,58]. In the present study, although BAC (0.2–0.6 µg/mL) treatment for 7–21 days induced EMT via the upregulation of EMT transcription factors in H358 cells, cell viability was not altered. It has been reported that non-toxic concentrations (0.5–0.75 µg/mL) of BAC elevated TGF-β1, TGF-β receptor1, Smad3, and phospho-Smad3 protein levels in conjunctival fibroblasts [59]. Since lung epithelial cells exposed to toxic stimuli can express TGF-β, which then promotes EMT by its autocrine action [27], the activation of the TGF-β signaling pathway could be a possible reason for BAC-promoted EMT in the present study. Moreover, ER stress by BAC (Figure 4) treatment could result in EMT due to ER stress inducers, such as tunicamycin and thapsigargin, in lung epithelial cells [60,61]. Previously, it has been reported that inhalation of BAC aerosol for 2 weeks caused pulmonary fibrosis in rats [15]. In the present study, long-term incubation of cells with BAC led to increased expression levels of ECM components, fibronectin, and vimentin (Figure 5 and Figure 6) suggesting the fibrogenic effect of BAC through EMT. Nevertheless, the role of BAC-promoted EMT in animal lung fibrosis is unclear.

## 5. Conclusions

In conclusion, we document, that BAC can induce necrosis, apoptosis, ER stress, and EMT in lung epithelial cells in a time- and concentration-dependent manner. These findings can contribute to the understanding of cellular responses in BAC-exposed lung epithelium, but the exact effects of this substance on the animal lung airway remain unclear. Therefore, further in vivo experiments such as rodent inhalation studies are required to clarify the apoptotic and EMT inducing effects of BAC in the pulmonary organs of animals.

## Figures and Tables

**Table 1:** Human primers used for real-time reverse transcription-polymerase chain reaction (RT-PCR).

Genes	Primer Sequences	
E-cadherin	F: TCCATTTCTTGGTCTACGCC	R: CACCTTCAGCCATCCTGTTT
N-cadherin	F: ACAGTGGCCACCTACAAAGG	R: TGATCCCTCAGGAACTGTCC
Fibronectin	F: TCGAGGAGGAAATTCCAATG	R: CTCTTCATGACGCTTGTGGA
Vimentin	F: GGCTCAGATTCAGGAACAGC	R: GCTTCAACGGCAAAGTTCTC
Snail	F: CCAGACCCACTCAGATGT	R: GCAGGTATGGAGAGGAAGA
Slug	F: CATCACTGTGTGGACTACC	R: CTTGGAGGAGGTGTCAGA
Twist	F: GCACCATCCTCACACCTC	R: CTGATTGGCACGACCTCT
Zeb1	F: GCACAACCAAGTGCAGAAGA	R: GAACCATTGGTGGTTGATCC
Zeb2	F: CAACTCCGATGAACTGCTGA	R: AGCCTGAGAGGAGGATCACA
18S	F: CAGCCACCCGAGATTGAGCA	R: TAGTAGCGACGGGCGGTGTG
